# The draft genome of the tropical sea cucumber *Stichopus monotuberculatus* (Echinodermata, Stichopodidae) reveals critical genes in fucosylated chondroitin sulfates biosynthetic pathway

**DOI:** 10.3389/fgene.2023.1182002

**Published:** 2023-05-12

**Authors:** Shengping Zhong, Xiaowan Ma, Yan Jiang, Xujia Liu, Mengqing Zeng, Longyan Zhao, Lianghua Huang, Guoqiang Huang, Yongzhen Zhao, Ying Qiao, Xiuli Chen

**Affiliations:** ^1^ Guangxi Key Laboratory of Marine Drugs, Institute of Marine Drugs, Guangxi University of Chinese Medicine, Nanning, China; ^2^ Guangxi Engineering Technology Research Center for Marine Aquaculture, Guangxi Institute of Oceanology Co., Ltd., Beihai, China; ^3^ Key Laboratory of Tropical Marine Ecosystem and Bioresource, Fourth Institute of Oceanography, Ministry of Natural Resources, Beihai, China; ^4^ Guangxi Key Laboratory of Marine Environmental Science, Guangxi Academy of Marine Sciences, Guangxi Academy of Sciences, Nanning, China; ^5^ Guangxi Key Laboratory of Aquatic Genetic Breeding and Healthy Aquaculture, Guangxi Academy of Fishery Sciences, Nanning, China

**Keywords:** *Stichopus monotuberculatus*, fucosylated chondroitin sulfates, biosynthetic pathway, genomic adaptation, Echinodermata

## Introduction

Holothuroids, or sea cucumbers, are a diverse echinoderm group of economically significant marine benthic invertebrates (Han et al., 2016; Miller et al., 2017). Due to its large bode size and thick body wall, *Stichopus monotuberculatus*, a tropical holothuroid has become a popular tonic food in Indo-west pacific counties, where it is known as its delectable taste and high nutritional value (Chen et al., 2022). Because the body walls of holothuroids contain abundant and diverse biomedical compounds, such as sulfated polysaccharides, saponins, and peptides (Thimmappa et al., 2022) they have become the subject of increasing attention in pharmaceutical research as an important source of medicinal compounds (Xu et al., 2022).

Marine sulfated polysaccharides usually occur in echinoderms and brown algae, which are abundant and considered to be promising sources of medicinal compounds with significant pharmacological effects (Helbert, 2017; Lu et al., 2020). The marine sulfated polysaccharides are typically composed of sulfate groups and *α*-L-fucose. They are structurally diverse and the positions of their fucosylated and sulfonated features vary by marine species (Pomin, 2014; Wu et al., 2016). Sulfated fucans (SF) and fucosylated chondroitin sulfates (FCS) are the two most common types of sulfated polysaccharides occurring in marine animals, with SF occurring in echinoderms and brown algae (Helbert, 2017) and FCS being exclusive to echinoderms (Carvalhal et al., 2019). Fucosylated chondroitin sulfates, also called fucosylated glycosaminoglycan (FG), is typically composed of a chondroitin sulfate backbone attached to *α*-L-fucose branches, both of which are commonly sulfonated (Wu et al., 2016; Khotimchenko, 2018). The structural features of FCS and the pattern of sulfonation of the chondroitin sulfate backbone and the *α*-L-fucose branches varies across sea cucumber species (Yuan et al., 2022). It be important in maintaining the body wall integrity of sea cucumbers by preventing degradation by marine microorganisms (Xu et al., 2022). Studies of brown algae have detected duplication and expansion events at SF biosynthesis associated genes, such as the mannuronan C-5-epimerases (MC5Es) (Ye et al., 2015) and carbohydrate sulfotransferases (CHSTs), which increase the rigidity of the cell wall and provide genomic adaptions to different environments (Lu et al., 2020). A previous study on the structural diversity of holothurian FCS and the position and number of the fucosylated branches attached to the chondroitin sulfate backbone confirmed the species-specific patterns across holothuroids (Myron et al., 2014). These species-specific FCS structures may have significant impacts on various biological activities such as antithrombotic, antihyperlipidemic, and anticoagulant processes and wound healing (Mou et al., 2018). Studies of brown algae genomes have identified genes coding for key enzymes for sulfated polysaccharides (Nishitsuji et al., 2016). Moreover, the genes coding for downstream enzymes, such as sulfotransferases and fucosyltransferases have been revealed expanded in brown algae genome (Nishitsuji et al., 2019). However, despite the biological, ecological and economic importance of holothurian FCS, the key genes associated with its biosynthetic pathways and their evolution remain unknown.

Here in this report, in order to decipher the key genes associated FCS biosynthetic pathways in tropical sea cucumbers, *S. monotuberculatus* genome have been assembled and annotated. The genome comprised 168 contigs, with an overall length of 839.56 Mb. The contig N50 length was 11.51 Mb. In addition, our investigations of the evolution of the gene families revealed 1,011 significantly expanded genes and 408 genes exclusive to *S. monotuberculatus*. The gene families coding for key enzymes associated with FCS biosynthetic pathways, including fucosyltransferases and sulfotransferases, were also significantly expanded. The accessibility of first genomic sequences and annotations of *S. monotuberculatus* will provide a genomic approach to investigate the structural diversity of holothurian FCS, as well as novel perspectives into evolutionary adaptation of critical genes in holothurian FCS biosynthesis pathways.

## Materials and methods

### DNA and RNA sequencing

An individual *S. monotuberculatus* (body weight 5.92 g) was collected from Guangxi Province, China (21.484,179 N, 109.720,886 E), and kept in an aerated water tank before tissue sampling. The intestine, tentacles, muscle, and tube-feet were dissected out and flash-frozen using liquid nitrogen. Genomic DNA was isolated from a muscle sample using a QIAamp DNA Mini Kit (QIAGEN, Hilden, Germany). A Nanopore 20 kb insert library was constructed using approximately 1 µg of genomic DNA and Blue Pippin electrophoresis (Sage Science, Inc., Beverly, CA, United States) was used to select DNA fractions larger than 20 kb. A DNA sequencing library for *S. monotuberculatus* was prepared by Grandomics Biosciences Co., Ltd (Wuhan, China) using an SQK-LSK109 ligation kit (Oxford NanoporeTechnologies, Oxford, UK). In addition, a 350 bp insert size paired-end DNA sequencing library with 2 × 150 bp lengths was prepared using a MGISEQ-2000 platform (Wuhan, China) in accordance with the manufacturer’s instructions. Finally, *S. monotuberculatus* RNA was isolated from the intestine, tentacle, and tube-feet samples using an RNAiso kit (TaKaRa, Tokyo, Japan) and then a MGISEQ-2000 system was used to prepare three *S. monotuberculatus* paired-end RNA sequencing libraries with lengths of 2 × 150 bp.

### Genome assembly

The 2 × 150 bp paired-end DNA sequences were used to evaluate the *S. monotuberculatus* genome length and polymorphisms. After using fastp (v. 0.23.2) (Chen et al., 2018) to trim the DNA sequences, Jellyfish (v. 2.3.0) (Marçais and Kingsford, 2011), and Genomescope (v, 2.0) (Ranallo-Benavidez et al., 2020) were used to conduct a k-mer analysis with a length setting of 19. The correct-then-assemble strategy of NextDenovo (v. 2.5.0) (github.com/Nextomics/NextDenovo) was used to assemble the preliminarily *S. monotuberculatus* genome using Nanopore sequencing data. NextPolish (v. 1.4.1) (Hu et al., 2020) was used to fix the contig errors in the preliminarily *S. monotuberculatus* genome using *S. monotuberculatus* paired-end DNA sequences. Purge_Dups (v. 1.2.6) (Guan et al., 2020) was then used to remove redundant sequences. Finally, the completeness of the *S. monotuberculatus* genome was assessed using the metazoa_odb10 data in the BUSCO software (v. 5.4.4) (Manni et al., 2021).

### Prediction of transposable elements, gene structure, and functional annotation

Initially, we used EDTA (v. 2.1.0) (Ou et al., 2019) to apply an *ab initio* strategy to evaluate the transposable elements (TEs) in the *S. monotuberculatus* genome. A homology-based strategy was then applied using the predicted TEs library and RepeatMasker (v. 4.1.2) (www.repeatmasker.org) to further detect any remaining comprehensive TEs sequences. The protein-coding gene structures were annotated using a combination of transcript-based, homology-based, and *ab initio* prediction strategies. For the transcript-based annotation, the RNA sequencing data from the intestine, tentacles, and tube-feet samples were used to assembly *de novo* transcript sequences using Trinity (v. 2.14) (Grabherr et al., 2011). Afterwards, the genome guided-transcripts were assembled using StringTie (v. 2.2.1) (Shumate et al., 2022). Finally, the transcript-based predicted proteins in the *S. monotuberculatus* genome were obtain by aligning all transcript sequences to the *S. monotuberculatus* genomic sequences using PASA (v. 2.5.2) (Campbell et al., 2006) with default parameters. For the homology-based method, the protein-coding genes in the *S. monotuberculatus* genome were validated using GeMoMa (v. 1.9) (Hoff, 2019) with default settings obtained from the echinoderm protein data in Genbank, including those for *Strongylocentrotus purpuratus* (GCF_000002235.5), *Patiria miniata* (GCF_015706575.1), *Lytechinus variegatus* (GCF_018143015.1), *Apostichopus japonicus* (GCF_002754855.1), *Holothuria glaberrima* (GCF_009936505.2), *Asterias rubens* (GCF_902459465.1), *Anneissia japonica* (GCF_011630105.1), and *Chiridota heheva* (GCF_020152595.1). For the *ab initio* method, the RNA sequencing data of *S. monotuberculatus* and the echinoderm protein data were applied to predict the coding genes using BRAKER2 (v. 2.1.6) (Brůna et al., 2021) in the GeneMark-ETP mode. Finally, the predictions of the three strategies were merged and evaluated using EvidenceModeler (v. 1.1.1) (Haas et al., 2008), and the evaluated results were functionally annotated using DIAMOND (v. 2.1.3) (Buchfink et al., 2021) with default parameters to search several protein databases, and well as the Gene Ontology (GO), Swiss-Prot, UniProtKB-TremBL, and Kyoto Encyclopedia of Genes and Genomes (KEGG) databases, using an E-value limit for homologous annotation of 1e-5. The completeness of the prediction of the protein-coding genes in the *S. monotuberculatus* genome was further validated using BUSCO (v. 5.4.4) with the metazoa_odb10 data. The identification of transfer RNAs (tRNAs) and ribosomal RNAs (rRNAs) in the *S. monotuberculatus* genome was accomplished using tRNAscan-SE (v. 2.0.6) (Lowe and Chan, 2016) and RNAmmer (v. 1.2) (Huang et al., 2009), respectively. The identification of microRNAs and small nuclear RNAs (snRNAs) was performed using Infernal (v. 1.1.2) (Nawrocki et al., 2009) to search the Rfam database with default parameters.

### Phylogenomics and gene family investigation

To investigate the comparative phylogenomics and gene families of *S. monotuberculatus*, OrthoFinder (v. 2.5.4) (Emms and Kelly, 2019) was used to verify the orthologous gene clusters of *S. monotuberculatus* and 14 related species including *Acanthaste planci* (GCF_001949145.1), *Hemicentrotus pulcherrimus* (GCF_003118195.1), *Holothuria scabra* (GCF_026123075.1), *Pisaster ochraceus* (GCF_010994315.2), *Plazaster borealis* (GCF_021014325.1), *Saccoglossus kowalevskii* (GCF_000003605.2), *S. purpuratus*, *P. miniata*, *L. variegatus*, *Anneissia japonicus*, *H. glaberrima*, *A. rubens*, *Apostichopus japonica*, and *C. heheva*. The protein sequences of the 14 related species were retrieved from Genbank and Echinobase (www.echinobase.org), and the longest protein sequence was chosen to identify the orthologous protein. To investigate the evolutionary status of *S. monotuberculatus*, the single-copy protein sequences were first obtained from the orthologous OrthoFinder results and then MUSCLE (v. 3.8.31) was used to align those sequences. The phylogenetic tree was constructed from the continuous super protein sequences combined from all of the aligned single-copy sequences using RAxML-avx (v. 8.2.9) (Stamatakis, 2014) under LG4M amino acid substitution model with 1,000 bootstrap replicates. The evolutionary times of *H. scabra* and *A. japonicus* were retrieved from the TimeTree website (www.timetree.org) and calibrated using the r8s software (v. 1.71) to evaluate the evolutionary time of *S. monotuberculatus* and the 14 closely related species. In order to evaluate the evolutionary history of the *S. monotuberculatus* gene families, the likelihood analysis method was performed using CAFE (v. 5.0) (Mendes et al., 2020) with *p <* 0.05. In addition, functional enrichment analyses, including KEGG and GO, were employed using TBtools (Chen et al., 2020) to evaluate the biological functions of the *S. monotuberculatus* species-specific and expanded gene families.

## Results and discussions

### Genome assembly

**FIGURE 1:**
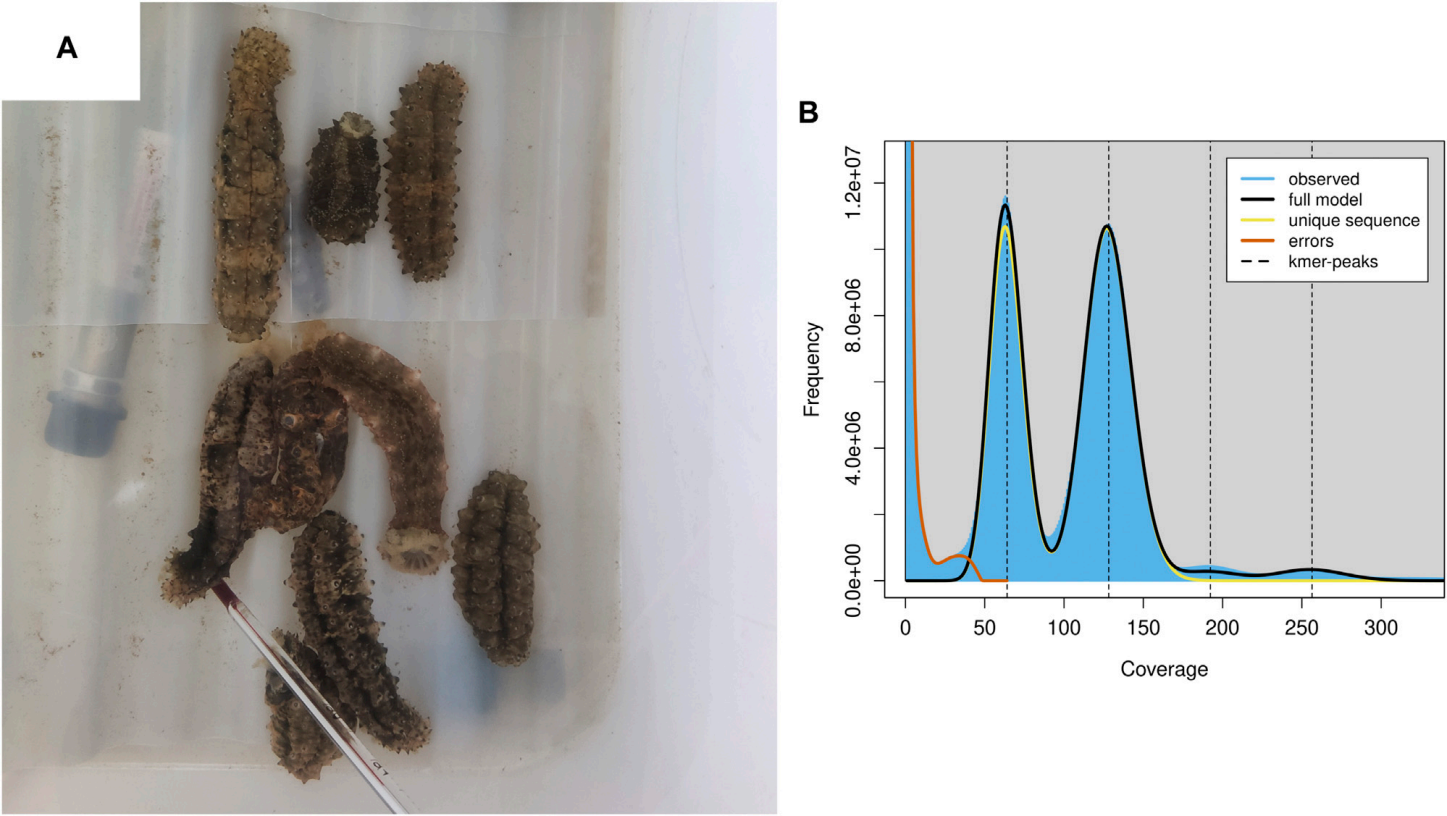
*S. monotuberculatus* and the genomics feature. **(A)** *S. monotuberculatus* reared in water tank. **(B)** The genomics feature analysis of *S. monotuberculatus*.

Both the Nanopore and MGI platforms were used to assemble the *S. monotuberculatus* genome. The former produced 51.53 Gb sequences with an average length of 22,615 bp, while the latter produced 64.72 Gb sequences with a Q20 quality level of 97.96%. The genomic features of the *S. monotuberculatus* genome were estimated using the MGI platform data in Genomescope to perform k-mer-based estimations. The results showed a predicted size of the *S. monotuberculatus* genome of 784.77 Mb, as well as a repetitive sequence ratio of 31.63% and a high heterozygous rate of 1.59% (Figure 1B; Supplementary Table S2). The heterozygous rate of the *S. monotuberculatus* genome was close to that of *A. japonicus*, while its genome size was slightly smaller (Zhang et al., 2017). Due to the high heterozygosity of the *S. monotuberculatus* genome, the long reads data were used in NextDenovo to assemble its complex regions. After a first round of assembly, 292 contigs with 10.52 Mb in contig N50 were achieved. To resolve the redundancy of heterozygous regions, Purge_Dups was applied after correcting contigs errors using NextPolish. Eventually, 168 contigs with 839.56 Mb in size, 11.51 Mb in contig N50, and a longest contig of 52.05 Mb were established in the *S. monotuberculatus* genome (Supplementary Table S1). This is the largest reported contig N50 for any holothurian genome to date (Luo et al., 2022). Based on the metazoa_odb10 lineages, the BUSCO analysis with genome mode was performed to assess the genome integrity of *S. monotuberculatus*. The result showed that it covered 97.9% BUSCOs, including 94.2% complete and 3.7% fragmented, while only 2.1% were classified as missing (Supplementary Table S3). Our assembled genome has the greatest level of completeness of any reported holothurian genome to date, including *H. scabra* (Luo et al., 2022), *H. glaberrima* (Medina-Feliciano et al., 2021), *C. heheva* (Zhang et al., 2022), and *A. japonicus* (Li et al., 2018). In addition, 95.98% of the short read data can be confidently mapped to the *S. monotuberculatus* final genome using BWA v. 0.7.17. These results indicate that the genome completeness and integrity are high in the final assembled *S. monotuberculatus* genome.

### Genome annotation

The integrated results of the predicted TEs analysis showed that a total length of 298.36 Mb (35.54%) of the *S. monotuberculatus* genome could be identified as TEs, which is the highest among the published Stichopodidae genomes, including *A. japonicus* (26.68%) and *Parastichopus parvimensis* (25.02%). However, the proportion of TEs in the *S. monotuberculatus* genome was much smaller than in other deep-sea holothurian genomes, such as *C. heheva* (56.40%) (Zhang et al., 2022) and *Paelopatides* sp*.* Yap (73.93%) (Shao et al., 2022). The *S. monotuberculatus* genome was found to be richest in terminal inverted repeats, which made up 19.41% of the genome sequences, while long terminal repeats (6.88%) and tandem repeats (5.67%) were the next two richest types (Supplementary Table S4). Using EvidenceModeler to combine protein-coding gene predictions, the *S. monotuberculatus* genome was confirmed to have 36,422 protein-coding genes (Supplementary Table S1). The protein-coding gene number of *S. monotuberculatus* was a bit higher than that of *A. japonicus* (30,305), but the protein-coding gene length of *S. monotuberculatus* (12,278 bp on average, Supplementary Table S1) was a bit longer than that of *A. japonicus* (7,787 bp on average) (Zhang et al., 2017). Compared to *S. monotuberculatus* and *A. japonicus*, *H. scabra* genome has a much longer protein-coding gene length (25,967 bp on average) but much less protein-coding gene number (16,642) (Luo et al., 2022). In the comparison study of three *A. japonicus* genome assemblies, Jo’s assembly has the lowest protein-coding gene number (21,771) and the shortest protein-coding gene length (5,388 bp on average). However, as the quality of the assembly has improved, the gene annotation completeness has also increased significantly, resulting in the longest protein-coding gene length (8,918 bp on average) and the highest protein-coding gene number around thirty-thousand (Li et al., 2018). The improvement of *S. monotuberculatus* assembly quality may also result in an increase in the number of predicted protein-coding genes. The BUSCO evaluation based on metazoa_odb10 lineages showed that the verified genes covered 99.1% of metazoa_odb10 genes, including 98.6% classified as complete and 0.5% classified as fragmented (Supplementary Table S3). The completeness of the gene predictions in the *S. monotuberculatus* genome is the greatest of any reported holothurian genome to date, indicating that the quality of the gene structure prediction is sufficiently high for downstream analyses, such as comparative phylogenomics analysis. In addition, 79.31% of the *S. monotuberculatus* genes were functionally annotated in the various protein databases, including UniProtKB-TremBL (79.08%), Swiss-Prot (49.96%), KEGG (38.25%), and GO (36.82%) (Supplementary Table S5). Moreover, a total length of 0.34 Mb in the *S. monotuberculatus* genome was predicted to be non-coding RNAs, including 1,101 tRNAs, 1,452 microRNAs, 181 rRNAs, and 208 snRNAs (Supplementary Table S6).

### Phylogenomics and gene family investigation

The orthologous genes shared between *S. monotuberculatus* and other Echinodermata species were identified using OrthoFinder. A total of 30,979 genes (85.1%) were identified as orthologous gene clusters in the *S. monotuberculatus* protein-coding gene set, of which 2,793 genes (7.7%) were found to be species-specific orthologous genes. In addition, all Echinodermata share 6,191 orthogroups, along with 422 single-copy orthogroups (Supplementary Table S7). RAxML used 73,344 amino acid sites of these single-copy orthogroups to build the Echinodermata phylogenetic tree with the hemichordate *S. kowalevskii* rooted as an outgroup species. The phylogenetic results showed that *S. monotuberculatus* initially clustered with *A. japonicus*, and that Stichopodidae and Holothuriidae were clustered as sister groups to form the Aspidochirotida clade. Apodida (*C. heheva*) and Aspidochirotida were clustered together within the Holothuroidea, with Apodida being the basal taxon of Holothuroidea (Supplementary Figure S1). Our phylogenetic results indicated that Stichopodidae and Holothuriidae have a close sister relationship within Aspidochirotida, and that Apodida occupies the basal branch in the class Holothuroidea. These results were consistent with the phylogenetic analyses of Holothuroidea inferred by morphological (KERR and KIM, 2008) and phylogenomic analyses (Zhang et al., 2022).

**FIGURE 2:**
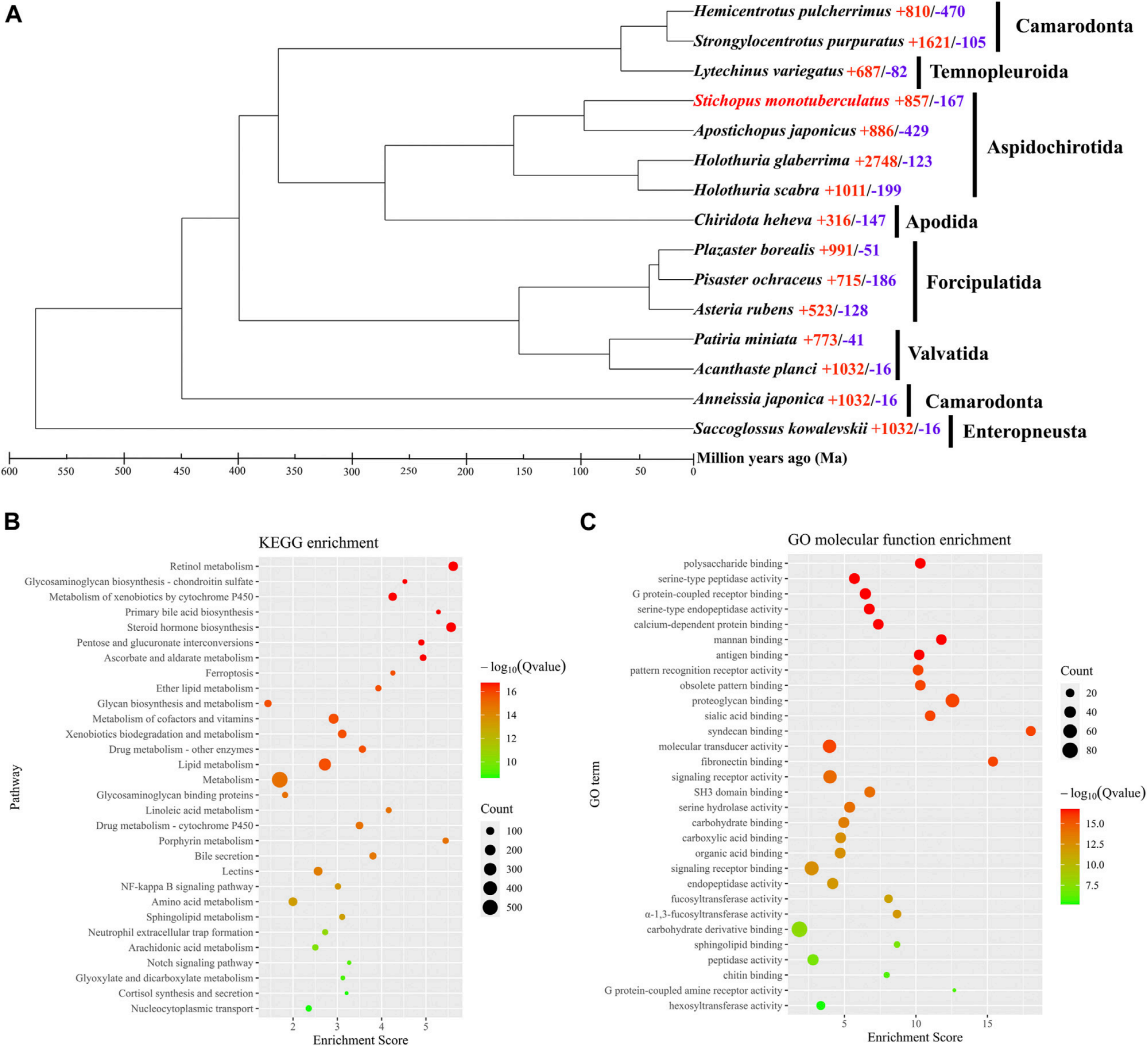
Phylogenomics and enrichment analyses. **(A)** Comparative phylogenomics analysis. Red numbers, indicates significantly expansion genes; while blue numbers, indicates significantly contracted genes. **(B)** KEGG enrichment analysis of significantly expansion genes. **(C)** GO enrichment analysis of species-specific genes.

An evolutionary investigation of gene families was conducted comparing *S. monotuberculatus* with other Echinodermata species. The results showed that 693 gene families in the *S. monotuberculatus* genome were unique and that 857 gene families had undergone significant expansion, whereas only 167 gene families had undergone significant contraction (Figure 2A). The structural features of FCS from holothuroids are species-specific, and mainly due to the different positions of the sulfonated branches on the chondroitin sulfate backbone and to the different fucosylated branch chains across the holothuroids (Xu et al., 2022). Studies of *S. monotuberculatus* have shown that these species-specific FCS structural features improve anticoagulant activity (Yuan et al., 2022). In this study, gene families coding for key enzymes involved in FCS biosynthesis, including carbohydrate sulfotransferases (CHSTs, EC 2.8.2) (Supplementary Figure S2) which were identified as significantly expanded gene families and fucosyltransferases (FUTs, EC 2.4.1) (Supplementary Figure S3) which were identified as species-specific to *S. monotuberculatus*. In previous studies on brown algae, the sulfotransferases and fucosyltransferases genes were also expanded independently, and the copies of these genes varied among brown algae genomes, which may be important in the efficient biosynthesis of SF (Nishitsuji et al., 2019). Compared with other Echinodermata species, 13 copies of the carbohydrate sulfotransferase 11 (CHST11, EC 2.8.2.5) gene, 10 copies of the carbohydrate sulfotransferase 13 (CHST13, EC 2.8.2.5) gene, and 22 copies of the carbohydrate sulfotransferase (CHST15, EC 2.8.2.33) gene were identified as significantly expanded in the *S. monotuberculatus* genome. Meanwhile, The KEGG enrichment analyses of significantly expanded *S. monotuberculatus* gene families showed that these genes were significantly enriched in 62 pathways, as well as those involved in FCS biosynthesis, such as Glycosaminoglycan biosynthesis - chondroitin sulfate, Pentose and glucuronate interconversions, Glycan biosynthesis and metabolism, and Glycosaminoglycan binding proteins (Figure 2B). Significant expansions of gene families coding for key enzymes associated with FCS biosynthesis in the *S. monotuberculatus* genome may play crucial roles in the biosynthesis of complex and diverse FCS and may be important for maintaining body wall integrity in *S. monotuberculatus*. Furthermore, eight copies of the fucosyltransferase 4 (FUT4, EC 2.4.1.-) gene, nine copies of the fucosyltransferase 7 (FUT7, EC 2.4.1.-) gene, and one copy of the fucosyltransferase 3 (FUT3, EC 2.4.1.65) gene were identified as unique to the *S. monotuberculatus* genome. In previous studies on brown algae, *N. decipiens* genome also has unique fucosyltransferase and extracellular matrix genes which may facilitate sulfated fucan biosynthesis in *Nemacystus decipiens* (Nishitsuji et al., 2019). Compared to other species of Aspidochirotida, these unique fucosyltransferase genes may be critical for *S. monotuberculatus* producing FCS more efficiently to maintain body wall integrity. GO enrichment analyses also showed that these unique genes were significantly enriched in the Glycosaminoglycan biosynthesis pathway, such as the polysaccharide binding (GO:0030247), proteoglycan binding (GO:0043394), fucosyltransferase activity (GO:0008417), and *α*-1,3-fucosyltransferase activity (GO:0046920) pathways (Figure 2C). In summary, the species specificity of key fucosyltransferase genes may play an important role in the biosynthesis of unique FCS in *S. monotuberculatus*, indicating the complex genomic adaptations for FCS biosynthesis in sea cucumbers.

## Data Availability

The datasets presented in this study can be found in online repositories. The names of the repository/repositories and accession number(s) can be found in the https://figshare.com/, https://doi.org/10.6084/m9.figshare.22177898.v1; https://www.ncbi.nlm.nih.gov/, PRJNA938157, SRR23615351, SRR23610520-SRR23610521 and SRR23604910-SRR23604915.
