# Supplementary material for: The draft genome of the tropical sea cucumber Stichopus monotuberculatus (Echinodermata, Stichopodidae) reveals critical genes in fucosylated chondroitin sulfates biosynthetic pathway
Source: Front Genet. 2023 May 12;14:1182002. doi: 10.3389/fgene.2023.1182002 (PMC10213396; doi:10.3389/fgene.2023.1182002)
Supplement: Supplementary file 1 [file Table1.docx]

**Supplementary Table 1.** Summary statistics of genome assembly and gene prediction of *S. monotuberculatus*.

| **Summary statistics of genome assembly** |  |
| --- | --- |
| Total length of genome (Mbp) | 839.56 |
| Contig N50 size (Mbp) | 11.51 |
| Contig number | 168 |
| The length of largest contig (Mbp) | 52.05 |
| Proportion of BUSCO in genome model (%) | 97.9 |
| **Summary statistics of gene prediction** |  |
| Protein-coding gene number | 36,422 |
| The length of largest protein-coding gene (bp) | 68,160 |
| Mean gene length (bp) | 12,278 |
| Mean transcript length (bp) | 1196 |
| Mean exons length (bp) | 199 |
| Mean exons number per gene | 6.0 |
| Proportion of BUSCO in proteins model (%) | 99.1 |

**Supplementary Table 2.** Estimation of genome size based on 19-mer statistics.

| Kmer | Depth | Genome size (Mb) | repeat content (%) | Heterozygous rate (%) |
| --- | --- | --- | --- | --- |
| 19 | 64.10 | 1465.55 | 31.63% | 1.59 |

**Supplementary Table 3.** The BUSCO result of *S. monotuberculatus* genome assembly and gene prediction.

|  | Term | Number | Ratio (%) |
| --- | --- | --- | --- |
| genome assembly | Complete BUSCOS (C) | 899 | 94.2 |
|  | Single-copy BUSCOS (S) | 891 | 93.4 |
|  | Duplicated BUSCOS (D) | 8 | 0.8 |
|  | Fragmented BUSCOS (F) | 35 | 3.7 |
|  | Missing BUSCOS (M) | 20 | 2.1 |
| gene prediction | Complete BUSCOS (C) | 941 | 98.6 |
|  | Single-copy BUSCOS (S) | 936 | 98.1 |
|  | Duplicated BUSCOS (D) | 5 | 0.5 |
|  | Fragmented BUSCOS (F) | 5 | 0.5 |
|  | Missing BUSCOS (M) | 8 | 0.9 |

**Supplementary Table 4.** Summary statistics for the annotated repeat sequences.

| Repeat Classes | Count | Length (bp) | % of genome |
| --- | --- | --- | --- |
| terminal inverted repeats | 502495 | 162864742 | 0.1941 |
| long terminal repeats | 137898 | 57803485 | 0.0688 |
| non-long terminal repeats | 11127 | 5550372 | 0.0066 |
| helitron | 81022 | 24559166 | 0.0293 |
| tandem repeats | 117276 | 47566137 | 0.0567 |
| Total | 849818 | 298362668 | 0.3554 |

**Supplementary Table 5.** Statistics for the functional annotation of protein-coding genes.

| **Database** | **Gene Number** | **Percent (%)** |
| --- | --- | --- |
| Swiss-Prot | 18,195 | 49.96 |
| TremBL | 28,801 | 79.08 |
| KEGG | 13,932 | 38.25 |
| GO | 13,411 | 36.82 |
| At least one database | 28,886 | 79.31 |
| Total | 36,422 | - |

**Supplementary Table 6.** Summary statistics of non-coding RNA annotation.

|  | **Type** | **Number** | **Average Length(bp)** | **Total Length(bp)** | **%Genome** |
| --- | --- | --- | --- | --- | --- |
| microRNAs | - | 1,452 | 83 | 120,067 | 0.0143 |
| tRNAs | - | 1,101 | 72 | 102,204 | 0.0122 |
| rRNAs | 5S | 161 | 114 | 18,348 | 0.0022 |
|  | 18S | 10 | 1844 | 18,444 | 0.0022 |
|  | 28S | 10 | 4694 | 46,942 | 0.0056 |
|  | Total | 181 | 463 | 83,734 | 0.0100 |
| snRNAs | CD-box | 23 | 110 | 2,541 | 0.0003 |
|  | HACA-box | 8 | 168 | 1,345 | 0.0002 |
|  | splicing | 177 | 152 | 26,938 | 0.0032 |
|  | Total | 208 | 148 | 30,824 | 0.0037 |

**Supplementary Table 7.** Summary statistics for orthogroups in *S. monotuberculatus* genome.

|  | *S. monotuberculatus* |
| --- | --- |
| Number of genes | 36422 |
| Number of genes in orthogroups | 30979 |
| Number of unassigned genes | 5443 |
| Percentage of genes in orthogroups (%) | 85.1 |
| Percentage of unassigned genes (%) | 14.9 |
| Number of orthogroups containing species | 14317 |
| Percentage of orthogroups containing species (%) | 46.3 |
| Number of species-specific orthogroups | 693 |
| Number of genes in species-specific orthogroups | 2793 |
| Percentage of genes in species-specific orthogroups (%) | 7.7 |
