# Supplementary material for: The draft genome of the tropical sea cucumber Stichopus monotuberculatus (Echinodermata, Stichopodidae) reveals critical genes in fucosylated chondroitin sulfates biosynthetic pathway
Source: Front Genet. 2023 May 12;14:1182002. doi: 10.3389/fgene.2023.1182002 (PMC10213396; doi:10.3389/fgene.2023.1182002)
Supplement: Supplementary file 2 [file Image1.pdf]

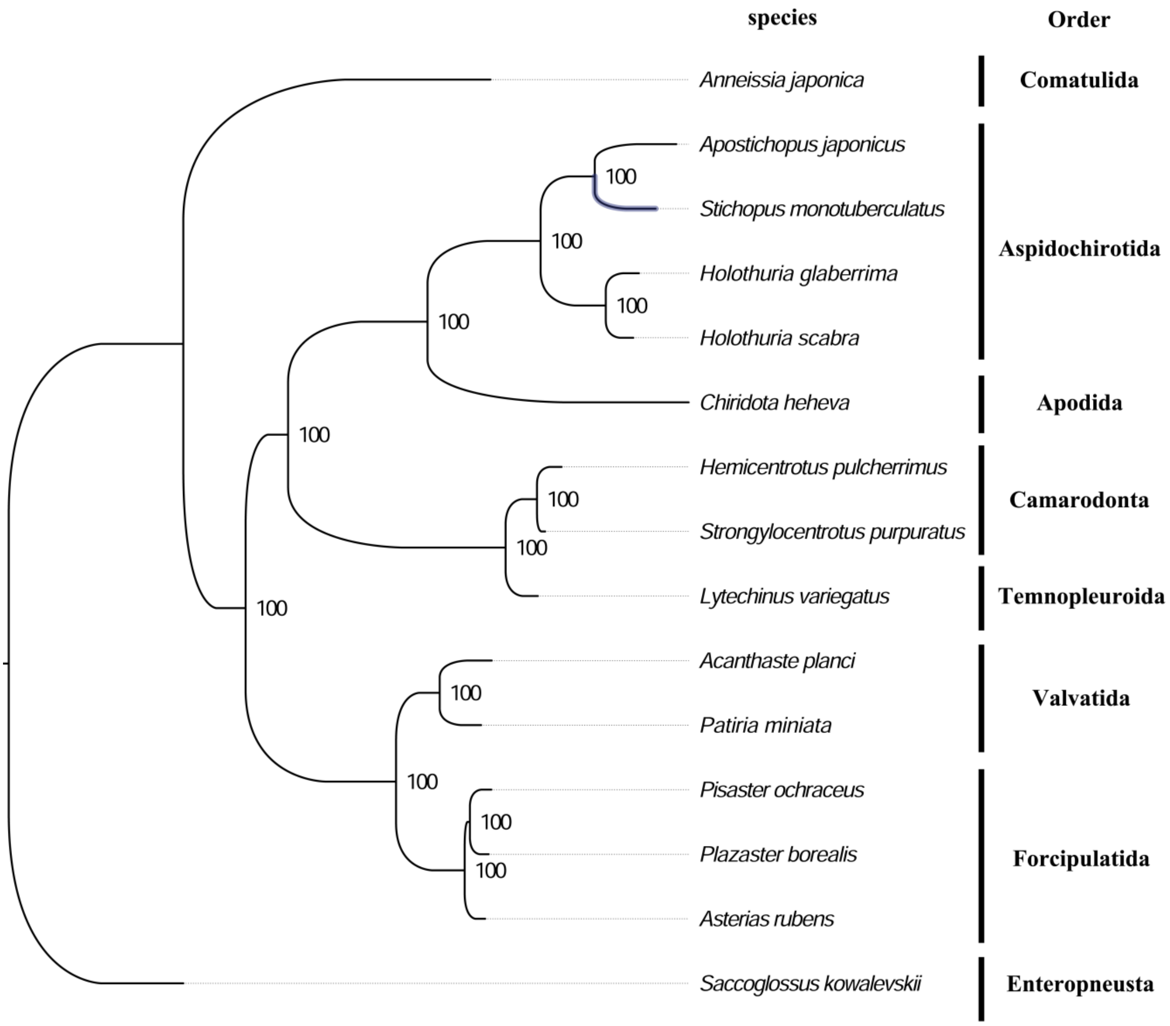

Supplementary Figure 1. Phylogenetic relationships between *S. monotuberculatus* and other Echinodermata species constructed with 422 single-copy orthologous using RAxML, the bootstrap values were labeled at each branch nodes.

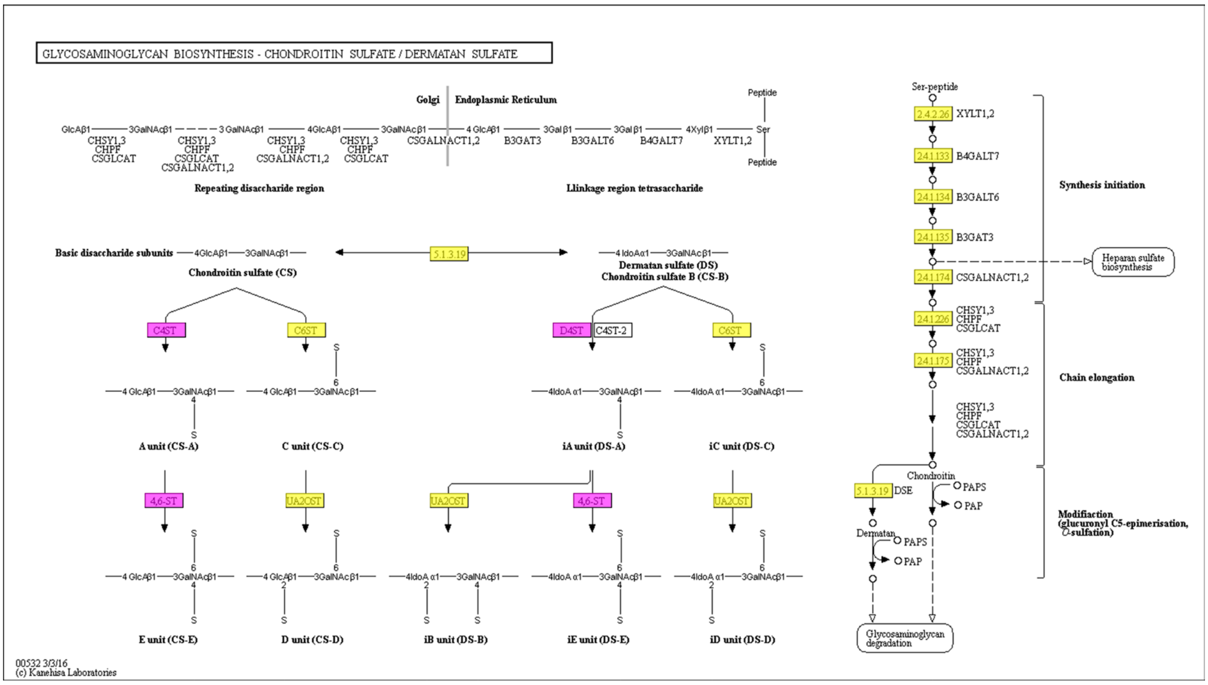

Supplementary Figure 2. Gene families list involved in glycosaminoglycan biosynthesis - chondroitin sulfate pathway. Pink, indicates significantly expansion gene families; and yellow, unchanged gene families.

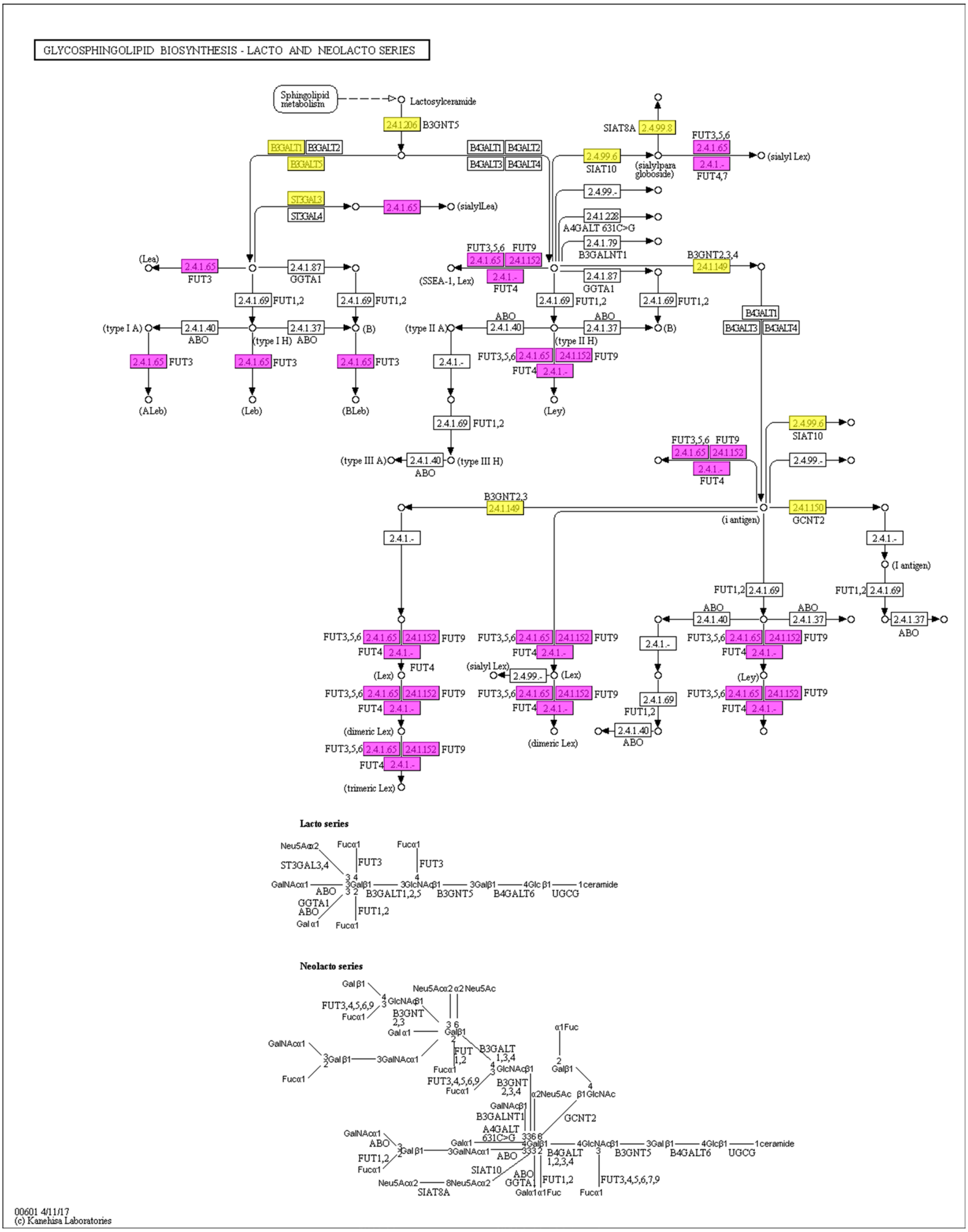

Supplementary Figure 3. Gene families list involved in Glycosphingolipid biosynthesis pathway. Pink, indicates species-specific gene families in *S. monotuberculatus* genome; and yellow, unchanged gene families.
